# Assessment of Blood Flow in Hepatocellular Carcinoma: Correlations of Computed Tomography Perfusion Imaging and Circulating Angiogenic Factors

**DOI:** 10.3390/ijms140917536

**Published:** 2013-08-27

**Authors:** Ya-Wen Chen, Huay-Ben Pan, Hui-Hwa Tseng, Yu-Ting Hung, Jer-Shyung Huang, Chen-Pin Chou

**Affiliations:** 1 National Institute of Cancer Research, National Health Research Institutes, Miaoli 350, Taiwan; E-Mail: ywc@nhri.org.tw; 2 Graduate Institute of Basic Medical Science, China Medical University, Taichung 404, Taiwan; 3 Department of Radiology, Kaohsiung Veterans General Hospital, Kaohsiung 813, Taiwan; E-Mails: hbpan@vghks.gov.tw (H.-B.P.); fatsesame@gmail.com (Y.-T.H.); jshuang@vghks.gov.tw (J.-S.H.); 4 Department of Medical Imaging and Radiological Sciences, I-Shou University, Kaohsiung 824, Taiwan; 5 School of Medicine, National Yang-Ming University, Taipei 112, Taiwan; E-Mail: hhtseng@vghks.gov.tw; 6 Department of Pathology, Kaohsiung Veterans General Hospital, Kaohsiung 813, Taiwan; 7 Department of Medical Laboratory Sciences and Biotechnology, Fooyin University, Kaohsiung 807, Taiwan

**Keywords:** hepatocellular carcinoma, blood flow, circulating angiogenic factors, CT perfusion, interleukin 8

## Abstract

Hepatocellular carcinoma (HCC) is a highly vascular tumor through the process of angiogenesis. To evaluate more non-invasive techniques for assessment of blood flow (BF) in HCC, this study examined the relationships between BF of HCC measured by computer tomography (CT) perfusion imaging and four circulating angiogenic factors in HCC patients. Interleukin 6 (IL-6), interleukin 8 (IL-8), vascular endothelial growth factor (VEGF), and platelet derived growth factor (PDGF) in plasma were measured using Bio-Plex multiplex immunoassay in 21 HCC patients and eight healthy controls. Circulating IL-6, IL-8 and VEGF showed higher concentrations in HCC patients than in controls (*p* < 0.05), and predicted HCC occurrence better than chance (*p* < 0.01). Twenty-one patients with HCC received 21-phase liver imaging using a 64-slice CT. Total BF, arterial BF, portal BF, arterial fraction (arterial BF/total BF) of the HCC and surrounding liver parenchyma, and HCC-parenchyma ratio were measured using a dual-vessel model. After analyzing the correlations between BF in HCC and four circulating angiogenic factors, we found that the HCC-parenchyma ratio of arterial BF showed a significantly positive correlation with the level of circulating IL-8 (*p* < 0.05). This circulating biomarker, IL-8, provides a non-invasive tool for assessment of BF in HCC.

## 1. Introduction

Hepatocellular carcinoma (HCC) is the third most common cause of cancer-related death worldwide with six million new cases diagnosed annually [1]. HCC is a highly vascular tumor, through the process of angiogenesis of the hepatic artery, as described by Folkman *et al*. [2–4]. HCC angiogenesis has been identified as being critical for tumor growth and metastasis [5–7]. Therefore, quantification of tumor angiogenesis or blood flow (BF) is important for risk stratification, disease progression, and response to cancer therapy [5,6]. Generally, quantification of HCC angiogenesis has been studied with magnetic resonance imaging (MRI) and computed tomography (CT) perfusion imaging, contrast ultrasound, Doppler ultrasound, microvessel density (MVD), and circulating biomarkers to correlate with the patient’s prognosis and response to therapy [5–8].

Clinical studies for the role of angiogenesis of HCC have mainly focused on CT perfusion parameters and histological examination of MVD using paraffin-embedded tissue specimens stained with specific endothelial cell markers and tissue expression of angiogenic molecules [9]. Histological observation of HCC angiogenesis is invasive and, therefore, impractical for repeat measurements. A minimally invasive method by CT perfusion requires high ionizing radiation doses and rapid bolus injection of iodinated contrast agent, which may cause rupturing of vascular walls and a life-threatening allergic reaction. Tissue contrast enhancement on CT perfusion imaging is based on arterial input function, progressive distribution of contrast agent into the small capillary, and leakage across the capillary walls to the extravascular extracellular space [6]. CT perfusion allows quantitative analysis of tumor BF and blood volume (BV) of HCC in clinical assessment. Perfusion CT, as a quantitative biomarker of angiogenesis, has been applied in studies of lung, colorectal, renal, and liver cancers [9–12]. The CT perfusion studies of HCC were performed by multidetector computed tomography (MDCT), based on the hepatic arterial supply method [13,14]. However, liver perfusion is more complicated than perfusion on other organs because the liver has dual-vessel flows [15]. Several recent studies have used more dedicated dual input (arterial/portal vein) CT perfusion to calculate the BF of HCC [16–23].

Growing evidence suggests the levels of circulating angiogenic factors might have significant implications in HCC patients [24]. The circulating angiogenic factors such as vascular endothelial growth factor (VEGF), interleukin 6 (IL-6), interleukin 8 (IL-8), and platelet-derived growth factor (PDGF) were positively correlated with tumor angiogenesis [25–28]. Significantly elevated in the serum of HCC patients, these circulating angiogenic factors were proposed as promising tumor markers for tumor progression [25–28]. Technically, multiple circulating angiogenic factors can be simultaneously analyzed using a recently developed multiplex immunoassay technique with a minimal amount of blood samples, providing a much faster test than conventional enzyme-linked immunosorbent assay (ELISA) techniques [29].

To our knowledge, there is still an equivocal relationship between BF of HCC and levels of circulating angiogenic factors in sera of HCC patients. BF in HCC has been reported to have a possibility for being a biomarker. However, currently, BF has not yet been established as a definitive biomarker of HCC [14]. To find convenient and quantitative parameters to evaluate BF of HCC in this study, we detected multiple circulating angiogenic factors by easily using a multiplex immunoassay technique and analyzed the BF of HCC performed by 64-slice CT perfusion imaging and commercial imaging software. Additionally, we compared the perfusion parameters between non-tumor liver parenchyma and HCC using a dedicated CT perfusion imaging with dual vascular data. Our data enforces us understand the relationship between CT perfusion parameters and circulating angiogenic factors and provides a non-invasive tool for assessment of BF in HCC.

## 2. Results

### 2.1. Circulating Angiogenic Factors in Patients with HCC and Controls

**Figure 1:**
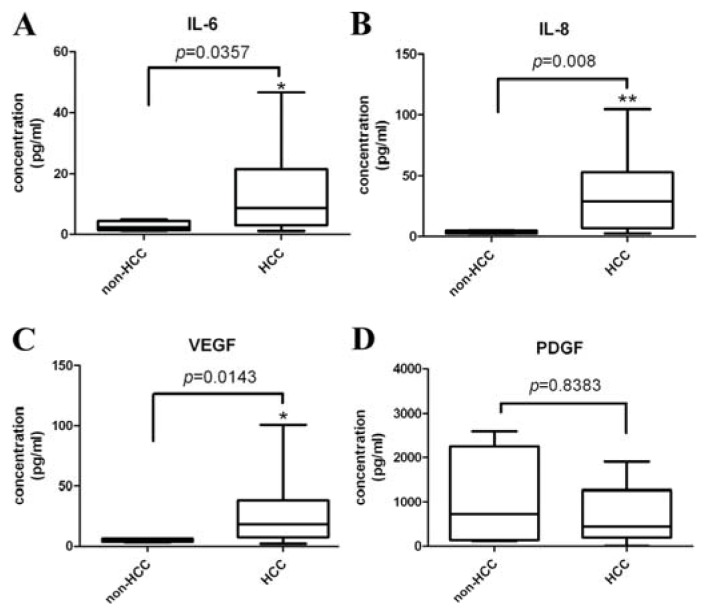
Descriptive statistics of (**A**) interleukin 6 (IL-6); (**B**) interleukin 8 (IL-8); (**C**) vascular endothelial growth factor (VEGF); and (**D**) platelet derived growth factor (PDGF) levels in the groups of HCC patients and healthy controls. Circulating angiogenic factors (IL-6, IL-8, and VEGF) were significantly higher in HCC patients than the control groups. *p < 0.05; **p < 0.01.

Circulating IL-6, IL-8, and VEGF were significantly higher in 21 HCC patients than eight healthy controls, (*p* < 0.05) (Figure 1A–C) but the PDGF did not significantly differ between these two groups (Figure 1D).

**Figure 2:**
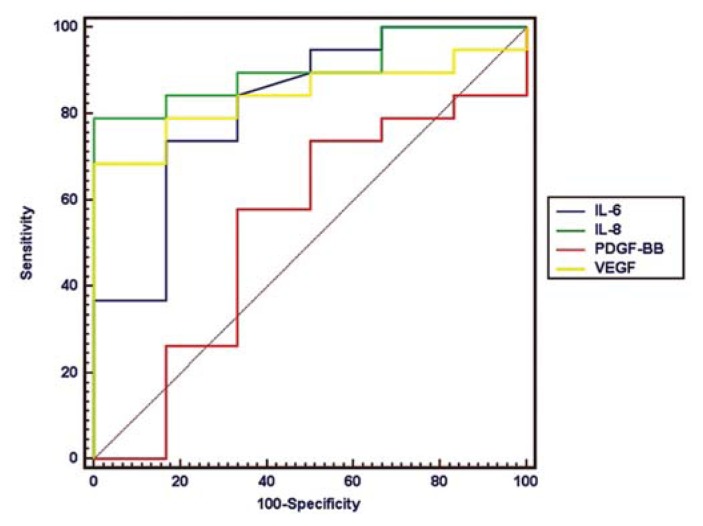
The diagnostic sensitivity and specificity of circulating angiogenic factors for HCC were assessed by receiver-operating characteristic (ROC) curves and the area under the ROC curve (AUR). The diagnostic performances of IL-8 (AUC, 0.865; sensitivity, 71%; specificity, 100%; cutoff value, 8.56 ng/L), VEGF (AUC, 0.842; sensitivity, 68%; specificity, 100%; cutoff value, 8.67 ng/L), and IL-6 (AUC, 0.79; sensitivity, 71%; specificity, 83%; cutoff value, 4.94 ng/L) in patients with HCC were better than chance (*p* < 0.01).

To evaluate the diagnostic applications of angiogenic factors for predicting patients with HCC, circulating IL-6, IL-8, VEGF, and PDGF were analyzed in HCC patients and controls using a receiver-operating characteristic (ROC) curve. The area under the curve (AUR) of IL-8, VEGF, IL-6, and PDGF were 0.865, 0.842, 0.79, and 0.532, respectively (Figure 2). The high AUR values of IL-6, IL-8, and VEGF indicated these three circulating angiogenic factors significantly differed from that of a chance alone result (AUR = 0.5) for its utility to discriminate HCC patients from healthy controls (*p* < 0.01). Circulating IL-8, IL-6, and VEGF also showed significantly better diagnostic performance than PDGF (*p* < 0.05).

### 2.2. CT Perfusion Parameters and Microvessel Density (MVD)

The average size of the target HCCs in 21 patients was 8.2 cm. The results of the CT perfusion parameters between HCCs and their surrounding liver parenchyma were shown in Table 1.

**Figure 3:**
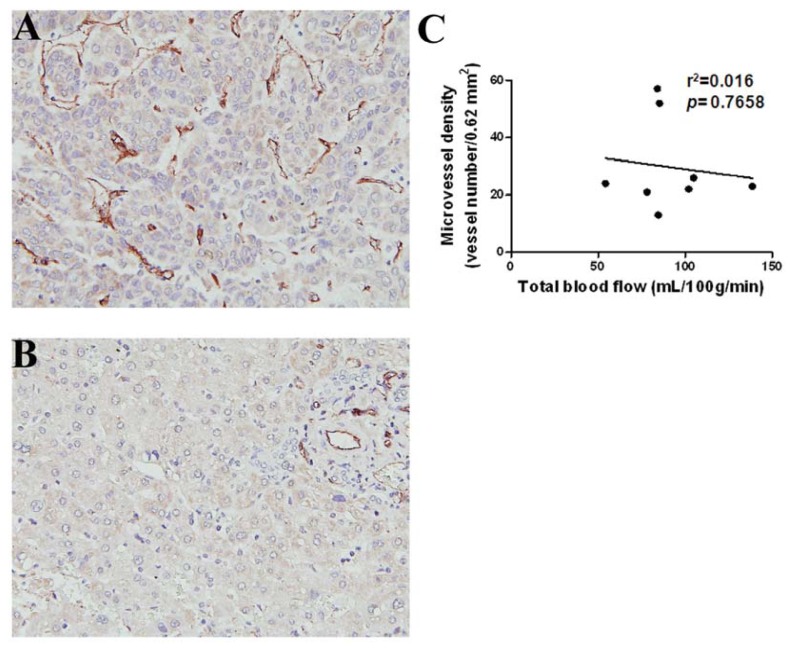
Histology and immunohistochemistry (IHC) of HCC and surrounding non-tumor liver parenchyma. (**A**) HCC section showed a higher numbers of CD31-positive endothelial cells (deep brown color) by IHC; (**B**) The section of the surrounding non-tumor region of liver parenchyma shows low CD31-positive endothelial cells by IHC; (**C**) Representative CT perfusion parameter (total BF) and average microvessel density (MVD), estimated by counting CD31-positive microvessels, did not show any significant trend. Microvessels were counted on a 200× field (0.62 mm^2^).

The measured region of interest (ROI) of HCCs showed higher arterial fraction (*p* < 0.001), lower portal BF (*p* < 0.001), and lower total BF (*p* = 0.002) than that of the surrounding liver parenchyma. There was no significant difference in arterial BF between the HCCs and surrounding liver parenchyma. These results suggested that BF in HCC was differentiated from that in the surrounding liver parenchyma. To examine if quantitative CT perfusion parameters was correlated with MVD, which was used as an indicator for evaluation of tumor angiogenesis, immunohistochemistry (IHC) with anti-CD31, a vascular marker, was conducted. The HCC sections with increased numbers of CD31-positive staining vessels in hot-spot regions demonstrated distinctly increased neovascularity compared to the surrounding liver parenchyma (Figure 3A,B). Following manual calculation, the MVD in HCCs (*n* = 8) did not show any significant correlation with the CT perfusion parameters (Table 2), such as total BF (Figure 3C).

### 2.3. Circulating Angiogenic Factors and CT Perfusion Parameters

**Figure 4:**
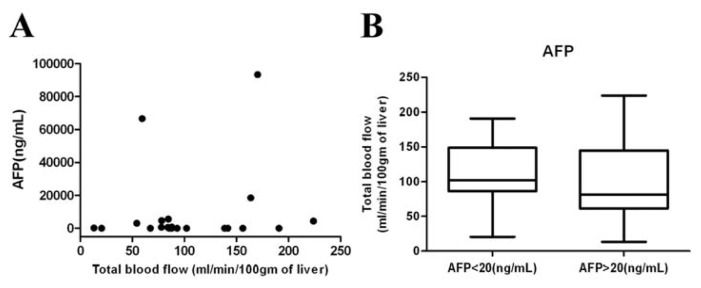
Relationships between alpha-fetoprotein (AFP) levels and total BF. (**A**) No correlation was detectable between plasma AFP and total BF, parameter of CT perfusion in HCCs (*r* = 0.2826, *p* = 0.2146); (**B**) Box plots of the total BFdistribution between patients with normal (AFP < 20 ng/mL, *n* = 9) and abnormal AFP (AFP > 20 ng/mL, *n* = 12). No significant difference was detected between the two groups of HCCs (*p* = 0.06).

After statistical analysis, none of the circulating angiogenesis factors were significantly correlated with any of the CT perfusion parameters, such as total BF, arterial BF, portal BF, and arterial fraction (Table 2). Additionally, α-fetoprotein (AFP), a serum marker for HCC detection, has been reported to correlate with MVD and expression of VEGF [30]. There was no significant correlation between the level of circulating AFP and CT perfusion parameters of HCCs, such as total BF (Figure 4A). No significant difference in CT perfusion parameters, such as total BF between HCC patients with low (<20 ng/mL, *n* = 9) or high AFP (>20 ng/mL, *n* = 12) in plasma level was found (Figure 4B).

### 2.4. HCC-Parenchyma Ratio of CT Perfusion Parameters and Circulating Angiogenic Factors

**Figure 5:**
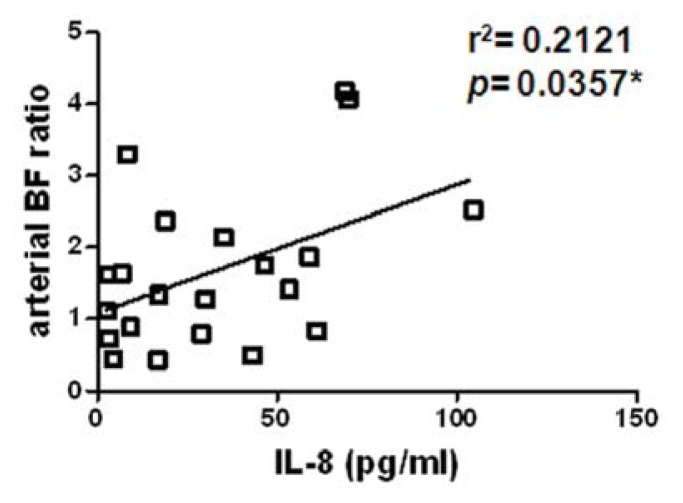
A correlation between the HCC-parenchyma ratio of arterial BF and level of circulating IL-8. There was a significant positive correlation (*r* = 0.46, *p* = 0.0357) between the HCC-parenchyma ratios of arterial BF and circulating IL-8. *p < 0.05.

To delicately measure the change in BF between HCC and its surrounding liver parenchyma, we calculated the HCC-parenchyma ratio (HCC/parenchyma) of the BF parameters. After analyzing, there was a significantly positive correlation between the HCC-parenchyma ratios of arterial BF and level of circulating IL-8, indicating a higher IL-8 level in circulation was associated with higher arterial BF in HCC relative to the surrounding parenchyma (Figure 5 and Table 2, *p* = 0.0357). However, there was no significant correlation between the HCC-parenchyma ratios of CT perfusion parameters and other factors, including IL-6, VEGF, and PDGF (Table 2).

## 3. Discussion

IL-8, a cytokine produced by macrophage, neutrophils, fibroblasts, and epithelial cells can directly affect vascular endothelial cell proliferation, matrix metalloproteinase production, vessel endothelial permeability, and tumor metastasis [28,31]. IL-6 is a significant mediator of the inflammatory response and involved in tumor angiogenesis and tumorigenesis [32,33]. The circulating IL-6 has higher diagnostic accuracy than serum AFP titers in HCC patients [25]. VEGF is a secreted cytokine that positively regulates tumor angiogenesis, and patients with HCC have higher level of circulating VEGF [26,34]. PDGF was released from hepatocytes and Kupffer cells in a hypoxia cirrhotic liver [35], and also plays a significant role in the process of angiogenesis and neovascular maturation [36]. The levels of circulating angiogenic factors distinguished between HCC patients and healthy controls in previous studies [25–28,37]. We also demonstrated circulating IL-6, IL-8, and VEGF had higher concentrations in HCC patients. Meanwhile, the elevated circulating IL-8 was also positively correlated with the increased HCC-parenchyma ratio of arterial BF, an indicator of BF in HCCs.

The primary difference between the previous studies and the current study is that we utilized multiplexed bead-based immunoassays for detecting the four circulating angiogenic factors in a clinical study. This assay has the advantages of a very small amount of blood sample needed, a single short diagnostic test, and multiplications for circulating proteins [29,38]. Similarly, our data indicated the circulating IL-6, VEGF, and IL-8 were sensitive and specific for predicting the occurrence of HCC among healthy controls and HCC patients [25,26,37]. However, we did not find significantly increased circulating PDGF in HCC patients.

The second difference in this study is that we used CT perfusion imaging by combining dedicated software with dual-vessel input of CT perfusion. The CT perfusion studies have been performed using single-vessel or dual-vessel inputs by different deconvolution-based analysis and kinetic models [13–23]. CT and MR perfusion parameters of HCCs and automatic selection of arterial input function could be obtained from maps generated using a dedicated software MIStar to evaluate BF of HCC [39]. CT perfusion parameters including total BF, arterial BF, and arterial fraction were analyzed from both arterial and portal venous enhancement references of HCC and the surrounding parenchyma. Previous studies have shown that CT perfusion is a feasible technique to assess the hemodynamic characteristics of HCC [9,13]. Wang *et al*. demonstrated BF in HCCs and surrounding liver parenchyma in a single-vessel CT perfusion model [40], which differs from our dual-vessel model. The blood supply of HCC is differently related to tumor size [41], tumor differentiation, and staging of HCC [4]. Since the portal vein takes a higher proportion of non-tumor liver perfusion than that of HCC, our method enrolled the information of artery and portal venous contribution, providing a better analysis between HCC and the surrounding liver parenchyma, as described in previous studies [16–23].

The third difference in this study is that we successfully correlated circulating angiogenic factors with *in vivo* CT perfusion imaging instead of *ex vivo* fixed tissue sections. The relationships between the imaging measurements of tumor BF have been investigated using MRI, CT, and Doppler ultrasonography [18,42,43]. Hsu *et al*. reported no significant relationships between the circulating VEGF level and the vascularity of HCCs on power Doppler ultrasonography following thalidomide therapy [44]. In this study, there was no correlation between the four angiogenic factors and the absolute values of BF measured by CT perfusion. The investigation of CT perfusion parameters of HCC could be more complicated in individuals. Thus, we further examined the HCC-parenchyma ratio of four CT perfusion parameters (total BF, arterial BF, portal BF, arterial fraction), which were calculated as the data of HCC divided by that of the surrounding liver parenchyma. We determined the HCC-parenchyma ratio because measurements in a ratio did eliminate some variables, such as the circulatory status in individuals. This resulted in different enhancement and body habitus in individual patients, and led to different measurements of attenuation of the X-ray beam and different Houndsfield unit values. Finally, we found levels of IL-8 had positive correlations with the HCC-parenchyma ratio of the arterial BF of the tumor. In contrast, we did not observe any correlation between IL-8 and MVD (*p* = 0.1046) or AFP (*p* = 0.1710). Our finding suggests that IL-8 is not only a detection marker of HCC but also correlated with BF of HCC.

We did not observe a significant correlation between the other three circulating angiogenic factors (IL-6, VEGF, PDGF) and BF measured by CT perfusion, even though IL-6 and VEGF did show good discrimination between patients with HCC and controls. Part of the reasons might be the relationships between circulating angiogenic factors and tumor angiogenesis may be a complex and dynamic process during HCC growth. Circulating IL-6 also plays a more important role in the tumorigenesis than angiogenesis of HCC [45]. Similar to the previous study [13], we did not find a significant difference in the BF of HCC in patients with low and high AFP levels. A recent study showed a negative correlation between increase in mean transit time (MTT) and AFP reductions after sorafenib therapy for advanced HCC [16]. However, their study did not find any significant correlation between other perfusion parameters (BF, BV, HAF, and PS) and AFP levels [16].

Similar to our data, the previous studies did not demonstrate a significant correlation between total BF and MVD [46,47]. One possible reason might be immature microvessels during tumor angiogenesis, and measured on histology MVD, usually present as very small tumor vessels, which would not reflect the total BF measured by CT perfusion imaging [6]. Vascular permeability is also an important factor in tumor angiogenesis because leakage of blood proteins from the circulation help to remodel the extravascular matrix for tumor vessel formation [48]. The CT perfusion parameter, measured by the contrast agent leakage from the intravascular space to extravascular extracellular space, reflects an *in vivo* dynamic microcirculatory blood volume than the counted vessels on paraffin-embedded tissue specimens. The disadvantage of direct pathologic analyses of angiogenesis included sampling error in heterogeneity distribution of tumor angiogenesis and invasive biopsy procedure. The contrast-enhanced CT technique is a easier and flexible method to evaluate the whole tumor than measuring MVD on limited specimens [49,50].

There are several limitations to our study. We only evaluated four of the most discussed circulating angiogenic factors of HCC, and other related circulating angiogenic factors were not examined. Increased circulating angiogenic factors may be released from peripheral blood, monocytes, liver fibrosis, or HCC [27]. The amount of different angiogenic factors among the liver parenchyma, HCC, and blood circulation require further clinical studies. We used the venous injection rate of 3.5 mL/s to avoid vessels rupture and leakage of contrast agent in this study. The model fitting is still adequate for the BF parameters, but the parameters of MTT would not be reliable. We did not discuss the parameter of MTT in this study, and used the arterial flow, arterial fraction, and total flow for analysis. Our CT scanning length, using 64-MDCT, is limited to a 3.2 cm, which only allowed the CT perfusion for studying partial tumor of large-sized HCC. Volume-rendered obtained with possible whole-liver perfusion CT for three-dimensional (3D) assessment of perfusion is still ongoing. For better visualization of HCC before contrast-enhanced CT scan, most of our patients had only one solitary large HCC, so data analysis from variable sized HCC are required in the future. There were a small number of HCC cases and controls in this study. Excess radiation exposure of CT perfusion limited the recruitment of more patients in our study and future clinical applications. However, our study has shown that the circulating IL-8 could be a feasible and noninvasive tool to examine BF in HCC to avoid ionizing radiation.

## 4. Experimental Section

### 4.1. Ethics Statement

This study was HIPAA compliant and approved by the institutional review board of Kaohsiung Veterans General Hospital, Taiwan (Protocol No. VGHKS97-CT6-07). Written informed consents were obtained.

### 4.2. Patient Selection

We conducted the dynamic contrast-enhanced CT study using a bolus of iodinated contrast agent in patients with HCCs. Patients having contraindications for contrast-enhanced CT examinations (contrast agent allergy, pregnancy), renal function impairment, poor localization of HCC on pre-contrast CT images, or refusing the CT perfusion exams were excluded from this study. Twenty-one patients with solitary HCC (*n* = 12) or multiple HCCs (*n* = 9), comprised of 19 men and 2 women with a mean age of 56 years (range 42–79 years), were enrolled in this study between 1 January 2009 and 1 December 2009. The diagnoses of HCC were based on histology (*n* = 8) or non-invasive criteria (*n* = 13) [51]. Patients underwent Child–Pugh classification for measuring acuteness of liver disease. Four groups included Category A (*n* = 14), Category B (*n* = 3), Category C (*n* = 2), and unknown category (*n* = 2). Among those 21 patients, one of them had metastases to lung and seven patients had lymph node metastases. Of the 21 patients, the additional imaging findings included liver necrosis (*n* = 7), irregular liver surface (*n* = 19), small peripheral branch portal vein thrombosis (*n* = 5), splenomegaly (*n* = 8), and recent HCC rupture (*n* = 2). The main portal vein remained patent and visible on CT perfusion in all patients on venous phase CT. Two patients presented with recurrent HCC after transarterial chemoembolization (TACE) or liver surgery. These enrolled patients had no local or systematic therapy at least six month before CT perfusion. One patient had hepatitis B Virus (HBV) and three had hepatitis C virus (HCV) infection. The peripheral blood of angiogenic factors and alpha-fetoprotein (AFP) from eight sex- and age-matched healthy controls (seven men and one woman, mean age 55 years) were also analyzed.

### 4.3. Measurement of Circulating Angiogenic Factors

On the same day of CT perfusion examination, 1 mL of blood was drawn from 21 HCC patients using a heparin rinsed syringe. The blood samples from the controls were also analyzed. The blood samples were prepared by centrifugation until the blood cells were separated from the plasma. The plasma was used for analyzing the angiogenic array performed using the microsphere-based Bio-Plex Suspension Array system (Bio-Rad Laboratories, Hercules, CA, USA). The data were finally analyzed using Bio-Plex Manager software (version 3.0, Bio-Rad: Hercules, CA, USA) with 5PL curve fitting.

### 4.4. CT Perfusion Imaging

**Figure 6:**
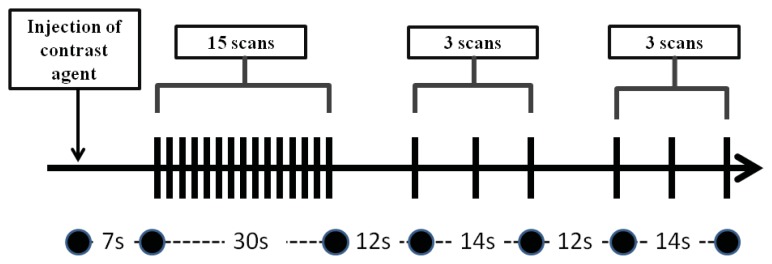
CT perfusion scanning protocol. Images are acquired at 21 time points.

The CT perfusion protocol was adapted from a previous liver perfusion study [52] using a 64-MDCT scanner (Aquilion 64, Toshiba Medical Systems Corporation, Otawara, Japan). For the initial localization of HCC, a pre-contrast CT scan of the entire liver was obtained with a 5 mm slice during a breath hold at the end of expiration. After the largest liver tumor was visualized on the non-enhanced CT images by an experienced abdominal radiologist (C.-P.C.) with 15 years of experience in liver imaging, a target 3.2-cm scanning length was selected for the CT perfusion study. The CT images were acquired in a single breath hold without table movement. A total of 70-mL of nonionic iodinated contrast agent (Isovue; Bracco, Princeton, NJ, USA) (300 mg of iodine/mL) was injected at a rate of 3.5 mL/s followed by saline flush using an 18-gauge intravenous cannula at the right elbow. The following CT parameters were used to acquire dynamic imaging data including: gantry rotation 400 ms; detector collimation = 1 mm × 64; 100 kVp, 240 mA, and 1 mm reconstructed slice thickness. The CT perfusion imaging was acquired using the established protocol [52]. Briefly, the images were acquired after a seven-second delay from the start of the injection, and 15 scans with an interval of two seconds were then acquired; six scans with an interval of seven seconds were then acquired twice. Images were acquired after a seven-second delay from the start of the injection, and 15 scans with an interval of two seconds were then acquired; three scans with an interval of seven seconds were then acquired twice. Intervals with durations of 12 s were taken between the 15th–16th and 18th–19th scans (Figure 6). Patients were asked to hold the breath during the CT scanning.

After CT perfusion imaging, the patients underwent a conventional venous phase imaging of the entire liver.

### 4.5. Post Processing Measurement of CT Perfusion

**Figure 7:**
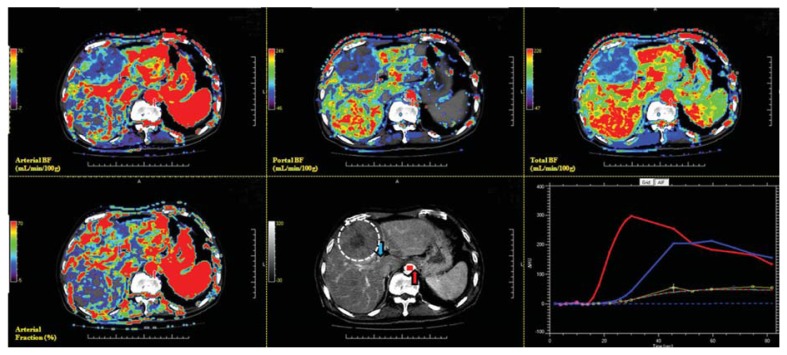
Screen display of a target HCC on MIstar workstation with full functional images and enhancing curve of HCC and liver parenchyma. CT functional maps include arterial BF (**upper left**), portal BF (**upper middle**), total BF (**upper right**), and arterial fraction (**lower left**). Semi-automatic determination of the artery (red arrow) and portal venous input (blue arrow) are illustrated in the lower middle panel. In the lower right panel, the red curve represented the enhancing change of the target artery. The blue curve represents the target portal vein, and the yellow curve indicates the region of interest (ROI) of the HCC.

Data on the CT perfusion of the target HCC among 21 patients were analyzed at a commercially available workstation (MIStar, Apollo Medical Imaging, Melbourne, Australia) by two experienced radiologists (J.-S.H. and C.-P.C.), specializing in liver imaging for more than 14 years. Region of interest (ROI) placement was determined based on the consensus of the two radiologists. The definitions of the CT perfusion parameters and the models were used for generating functional CT perfusion maps. The commonly used two-compartment model regards the extracellular space and plasma as two compartments that are individually well mixed [53]. The Materne-van Beers Liver Model, including singular deconvolution analysis of the hepatic artery and portal flow, will be used in data analysis [53]. HCC vascular functional maps of total BF, arterial BF, portal BF, and arterial fraction were generated on a workstation (Figure 7). BF measured with CT perfusion imaging reflects the intratumoral BF supplied by the total dual BF (sum of arterial BF and portal BF), hepatic artery (arterial BF), portal vein (portal BF), and arterial fraction (arterial BF/total BF).

**Figure 8:**
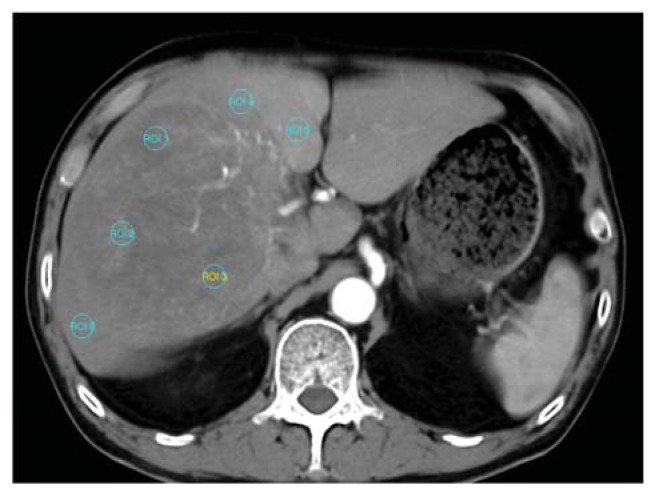
Six similar-sized region of interest (ROI) areas (1 mm^2^) are shown for measuring the CT perfusion BF of target HCC (ROI 1–3) and the adjacent non-tumor liver parenchyma (ROI 4–6).

Functional maps were obtained by (a) performing motion correction; (b) adjusting an appropriate window level for the liver vessels; (c) selecting sections between the beginning and end of contrast enhancement in the aorta and portal vein automatically or manually; (d) obtaining the reference arterial and portal venous input curve by placing an ROI in the aorta and portal vein respectively; (e) generating color-coded functional maps of CT perfusion; (f) drawing three similar-sized ROI areas (1 mm^2^) for each of the selected target HCC and surrounding non-tumor liver parenchyma (Figure 8); and (g) calculating the average perfusion parameters at an ROI region. The operators avoided areas of necrosis in the tumor and kept a distance from the margins of the tumor nodule to avoid partial volume effects. For tumor and liver parenchyma measurement, the operators kept out of ROI observed vessels filled with contrast agent. The size of the ROI remained constant in different measurements and in different patients. To compare the relative difference of vascularity between HCC and surrounding liver parenchyma, we measured the average CT perfusion parameters of HCC relative to background non-tumor liver parenchyma and calculated the ratio of BF parameters between the HCC and liver parenchyma (HCC-parenchyma ratio). The HCC-parenchyma ratios of BF were calculated using the BF data of HCC divided by that of the surrounding liver parenchyma.

### 4.6. Immunohistochemistry (IHC) and Tumor Vascularity Counting

To demonstrate the relationship between the CT perfusion parameters and vascular density measurement of HCC, we performed IHC of HCC tissue slides in eight patients for CD31 staining, which has been considered as a method for histologically quantifying small blood vessels. All tissue slides from HCC tumors were examined by hematoxylin and eosin (H & E) staining. All 5-μm-thick sections were deparaffinized and rehydrated. After antigen retrieval, the slides were blocked and then incubated with anti-CD31 Ab (1:200, rabbit polyclonal, Abcam, Cambridge, MA, USA). All of the tumor slides were observed under light microscopy at low magnification (×40) for any CD31-highlighted endothelial cells. The counting field was captured with a digital camera at five regions of the predominantly vascular areas (hot spots) under a high-power field (×200, 0.62 mm^2^ per field). MVD was presented as the average of CD31-positive countable blood vessels in five hot-spot areas [54].

### 4.7. Statistical Analysis

The ROC curve analysis was performed to examine the diagnostic sensitivity, specificity, and best cutoff values of the circulating angiogenic factors in the HCC patients and controls [42]. The ROC analysis performance was quantified by computing an AUR. An AUR of 1 indicated excellent diagnostic performance, while 0.5 indicated a performance no better than chance. Pairwise comparisons of AUR for various angiogenic factors were performed. We analyzed the correlations between two factors using Pearson r test or linear regression. We analyzed the significant difference between two groups using the Mann–Whitney U test, student *t* test, and Wilcoxon signed-rank test. Statistical calculation was performed using statistical software (SPSS, version 12.0; SPSS: Chicago, IL, USA; Medcalc version 9.3.6.0; MedCalc Software: Mariakerke, Belgium; GraphPad Prism, version 3.02; GraphPad Software: San Diego, CA, USA). Findings with a *p*-value of less than 0.05 were considered statistically significant.

## 5. Conclusions

The three circulating angiogenic factors (IL-6, IL-8, and VEGF) could predict HCC existence by a simple multiplex bead array assay. The circulating IL-8 can be an alternative biomarker for non-invasive evaluations of BF in HCC based on the evidence of this study.

## Figures and Tables

**Table 1:** Comparisons of computered tomography (CT) perfusion parameters between hepatocellular carcinomas (HCCs) and their surrounding non-tumor liver parenchymas.

CT perfusion parameters	HCC	Non-tumor parenchyma	Wilcoxon signed-rank test
Total BF (mL/min/100 g)	100.13 ± 33.69	156.81 ± 67.55	*p* = 0.002 **
Arterial BF (mL/min/100 g)	56.67 ± 20.06	47.62 ± 30.93	*p* = 0.10
Portal BF (mL/min/100 g)	42.72 ± 28.33	106.33 ± 70.07	*p* < 0.001 ***
Arterial fraction (Arterial BF/Total BF) (%)	57.99 ± 14.77	36.09 ± 20.67	*p* < 0.001 ***

Data are presented as mean ± standard derivation.

** *p* < 0.01,

*** *p* < 0.001.

BF: blood flow.

**Table 2:** Comparisons of computered tomography (CT) perfusion parameters with levels of angiogenic factors, microvessel density (MVD), and α-fetoprotein (AFP).

CT perfusion parameters	Angiogenic factors #					
*p* value	IL-6	IL-8	VEGF	PDGF	MVD #	AFP #
Total BF	0.8191	0.7788	0.8353	0.6820	0.7658	0.2146
Arterial BF	0.7864	0.7558	0.3398	0.0557	0.8396	0.7104
Portal BF	0.9357	0.5805	0.8837	0.4238	0.8702	0.2278
Arterial fraction	0.3227	0.8731	0.2239	0.1044	0.5364	0.5758
HCC-parenchymal ratio of total BF	0.9267	0.1520	0.9313	0.7516	0.5727	0.0029 **
HCC-parenchymal ratio of arterial BF	0.8009	0.0357 *	0.7571	0.0766	0.0678	0.0388 *
HCC-parenchymal ratio of portal BF	0.8722	0.5982	0.9482	0.7022	0.5616	0.1138
HCC-parenchymal ratio of arterial fraction	0.3368	0.0792	0.7441	0.2174	0.2278	0.4538

# Data were demonstrated using Pearson *r* test;

* *p* < 0.05;

** *p* <0.01.
